# Anterior Dislocation of the Tibiofibular Joint: A Case Report

**DOI:** 10.7759/cureus.37780

**Published:** 2023-04-18

**Authors:** Daniel Gonzalez-Arroyave, Mateo Arango Duque, Felipe Carrasco Velez, Harold Corrales Herrera, Carlos M Ardila

**Affiliations:** 1 Transplant, Hospital San Vicente, Medellin, COL; 2 Emergency, Suramericana Health Provider, Medellin, COL; 3 Orthopaedics, Universidad de Antioquia, Medellin, COL; 4 Basic Sciences, University of Antioquia, Medellin, COL

**Keywords:** computer tomography, treatment, diagnostic imaging, tibiofibular joints, knee joint

## Abstract

Dislocation of the proximal tibiofibular joint (PTJ) is a knee injury that occurs infrequently. In this case, the dislocation of the PJT of the right knee was reported with subsequent pain and limitation in range of motion, caused by trauma during the practice of a soccer game. An intense pain was observed in the area where the head of the fibula is located without finding crepitation or deformity. Initially, comparative anteroposterior and lateral X-rays of the knees were requested, showing proximal tibiofibular joint incongruity with anterolateral displacement without evidence of fracture lines. For this reason, it was decided to take a tomography of the right knee that confirmed the presence of anterior dislocation of the proximal tibiofibular joint. Closed reduction under sedation was scheduled.

## Introduction

The proximal tibiofibular joint has been classified as a synovial joint surrounded by a fibrous capsule that provides stability primarily with anterior, superior, and posterosuperior ligaments, plus additional support by an interosseous membrane, popliteal tendon, and fibular collateral ligament [1]. Dislocation of the proximal tibiofibular joint (DPTJ) is associated with less than 1% of knee injuries and is related to the practice of sports that involve violent movement of rotation and flexion of the knee [2]. It occurs most commonly in patients of approximately 25 years of age. It is often overlooked and underdiagnosed [3]. The instability of this joint can present with symptoms such as pain on the lateral side of the knee, discomfort when performing physical activity, or symptoms related to irritation of the common peroneal nerve [4]. Taking anteroposterior and lateral knee X-rays is not enough to make the diagnosis. When a lesion is suspected, the image of choice is computed tomography (CT) [1]. It has been found that on some occasions DPTJ can be spontaneous, but if this is not the case, the closed reduction under sedation has been proposed as a conservative treatment, and in some cases of late diagnosis, internal fixation or arthrodesis has been proposed as a solution, although the reports on this are scarce [4-6].

This case presents the diagnosis and treatment of this rare knee pathology.

## Case presentation

**Figure 1:**
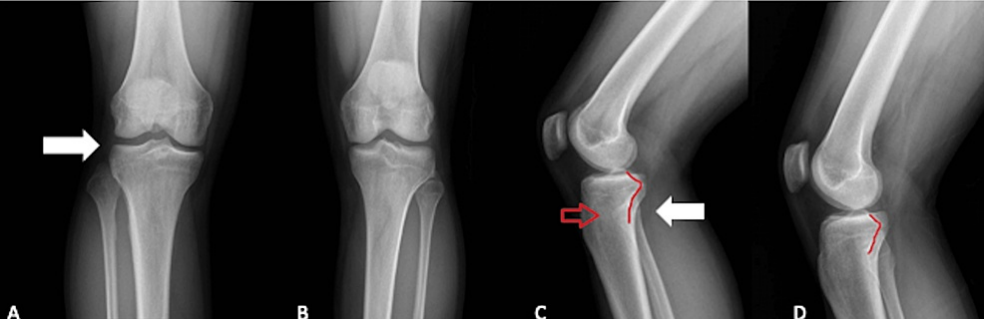
Anteroposterior radiograph of the right knee (A) showing decreased proximal tibiofibular overlap (arrow) compared to the contralateral side (B). Image C shows a lateral projection of the right knee, showing the fibular head (red arrow) anterior to Resnick's line (red line), compared to the contralateral side (D). Moreover, oblique morphology of both articular facets is observed (white arrow).

A 29-year-old male patient presented to the hospital due to symptoms of one hour's evolution with compromised rotational trauma in the right knee with subsequent pain in the lateral region and limited range of motion without erythema, edema, or deformity. During the evaluation, the patient reported trauma playing soccer. The pain was observed on palpation in the area where the head of the fibula is located, without finding crepitation or deformity. The patient only presented pain in the joint line in the lateral area; the neurological physical examination of the right lower extremity documented preserved plantar flexor muscle strength, without altered sensitivity of the fingers. Anteroposterior and lateral X-rays of comparative knees were requested, showing findings compatible with proximal tibiofibular joint incongruity, with anterolateral displacement, without evidence of fracture lines (Figure 1).

Given the doubts about the diagnosis, it was decided to complement the radiographs with a CT scan of the right knee, confirming the presence of a dislocation of the proximal tibiofibular joint of the right knee without compromising of the peroneal nerve.

**Figure 2:**
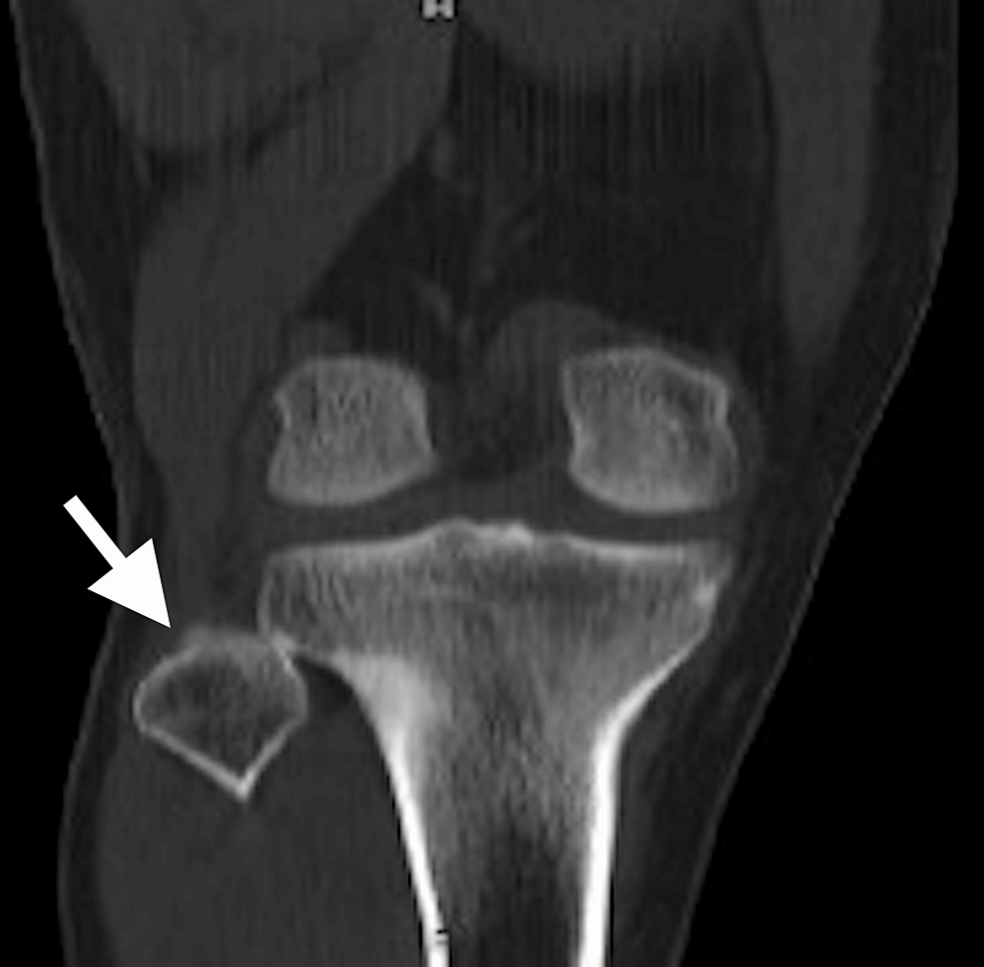
Tomography of the right knee (coronal section). Proximal tibiofibular joint incongruity with lateral displacement is evident (arrow).

**Figure 3:**
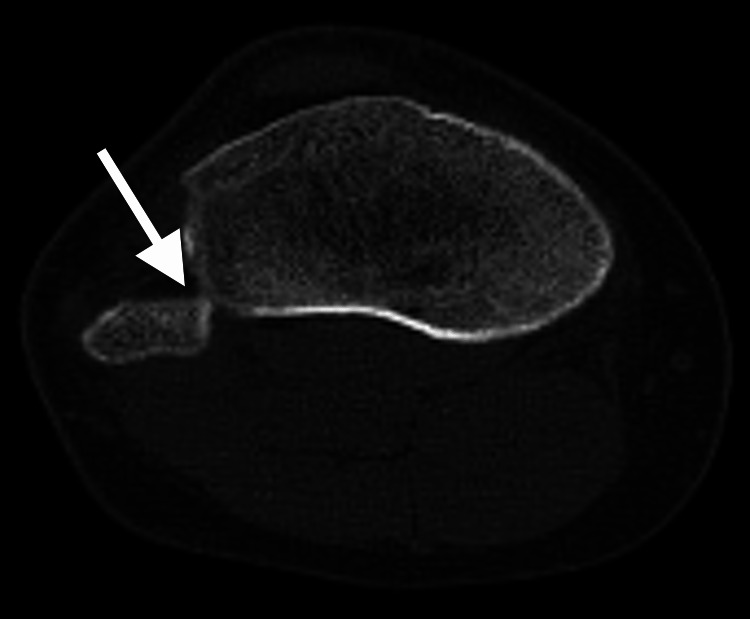
Tomography of the right knee (axial section). Evidence of proximal tibiofibular joint incongruity with anterior displacement (arrow).

The CT confirmed the presence of anterior dislocation of the proximal tibiofibular joint (Figures 2-3).

It was decided to perform closed reduction under sedation of the proximal tibiofibular joint. The patient was placed in a supine position with the right knee flexed to 90 degrees, the ankle plantigrade, and the foot in a neutral position. The examiner's non-dominant hand was placed on the medial edge of the knee with the dominant hand on the prominence of the fibular head, pressing it downwards and medially, until hearing and feeling a click (clunk). Improvement was observed in the lateral contour of the right knee. After sedation, the patient reported a complete absence of pain, improvement in limitation, and no documented nervous disturbance. The manual stability of the joint was evaluated by mobilization with the fingers, finding stability after reduction. The procedure was carried out successfully without complications and immobilization was performed with a Robert Jones-type bandage. Subsequently, he was discharged from the hospital with adequate analgesia. Restricted support with the use of crutches was recommended for three weeks and a follow-up appointment with orthopedics in three months. The patient did not attend this consultation; however, telephone follow-up was carried out at six and 11 months after the trauma and the patient reported complete resumption of his daily activities and return to sports activities at four weeks, with the same baseline level prior to the injury.

## Discussion

The proximal tibiofibular joint (PTJ) has been classified as a synovial joint, surrounded by a fibrous capsule that provides stability primarily by anterior superior and posterosuperior ligaments, with additional support by an interosseous membrane, popliteal tendon, and fibular collateral ligament [1]. However, with respect to bone morphology, two joint patterns have been described that are closely associated with joint stability. In his classic work, Ogden described horizontal facet morphology when the relationship between the inclination of the facet of the fibular head with respect to the horizontal axis is less than 20 degrees, which is considered more stable. On the other hand, oblique morphology can also be observed when this relationship between the facet and the horizontal axis is greater than 20 degrees, a relationship that is mostly associated with PTJ instability [2]. This finding was evidenced bilaterally in the lateral radiographs taken from the patient in the present case (Figures 1C-1D).

Isolated dislocation of the PTJ corresponds to less than 1% of knee injuries and is related to the practice of sports that involve violent movement of rotation and flexion of the knee, secondary to indirect trauma with foot inversion and plantar flexion [2]. Moreover, a second mechanism is found that consists of high-energy traumatic injuries that usually cause dislocations of the PTJ in conjunction with other injuries such as tibial fracture [7,8]. According to the clinical history of the patient presented in this case, it is possible to establish a direct relationship between the sports trauma suffered by the patient and the onset of symptoms, in addition to the faceted morphology of the joint, as elements causing the injury.

DPTJ is classified in the literature according to the case series presented by Ogden in 1974 where four types of patterns are presented: (1) joint subluxation established in immature skeletons and mainly women; (2) anterolateral dislocation secondary to indirect trauma; (3) posteromedial dislocation usually secondary to high-energy trauma and with a high probability of common peroneal nerve injury and (4) superior dislocation of the fibula [9].

The second type of lesion occurs more commonly in young patients. It is frequently overlooked and underdiagnosed [3]. Instability of this joint can present with symptoms such as lateral knee pain (most frequently reported) [10-13], which is exacerbated by palpation of the fibular head and ankle mobility [11], protrusion of the fibular head, discomfort during physical activity, or symptoms related to irritation of the common peroneal nerve [4].

Anteroposterior and lateral knee X-rays are the initial diagnostic images preferably requested. These have an additional benefit when done comparatively. Anatomical landmarks that may be useful have been described, such as Resnick's line, which is a radiopaque line that corresponds to the posterior surface of the lateral condyle of the tibia. This line should cut the fibular head in half, additionally, in the anteroposterior projection, an increase in the interosseous separation and loss of superposition between the tibia and fibula in its proximal portion can be seen [9-13]. When the lesion is suspected, the image of choice is CT [1]. In the case of this patient, conventional anteroposterior and lateral radiographs of the comparative knee were taken wherein the previously described findings were found, such as the location of the fibular head anterior to Resnick's line and the decrease in the proximal tibiofibular superposition; however, given the doubts about the diagnosis, it was decided to perform a simple CT of the right knee (Figure 2-3) to confirm or rule out the diagnostic suspicion, finding that in the simple radiographs, the findings were compatible with an Ogden-type proximal tibiofibular dislocation II, which was consistent with the clinical history and the finding on physical examination; however, it did not agree with the axial image in the CT scan, a finding for which no explanation was found in the literature. Unfortunately, no contralateral CT was performed to explain a possible anatomical variable, or if, on the contrary, a purely lateral dislocation would be considered, which has not been described in the literature.

Due to heterogeneity and scant evidence, there is currently no consensus on the definitive study and management of DPTJ [7]. As described in this case, most authors agree that clinical suspicion must be thorough and be confirmed with images, starting with radiography, and complementing with CT if suspicion persists [8,9]. However, magnetic resonance can also be helpful in differential diagnosis or in chronic cases [7].

Treatment options have been reported to be diverse depending on the acute or chronic stage diagnosis. Case reports have been found in which the dislocation is reduced spontaneously; however, there are other elements within the therapeutic tools available, such as closed reduction under sedation, and in some cases of late diagnosis, internal fixation or arthrodesis has been proposed as a solution, although reports on this are scarce [4-6].

There is general consensus that the treatment of DPTJ should begin with an attempt at closed reduction under sedation or local anesthesia, performed with the knee between 90 and 100° of flexion, with the ankle externally rotated and dorsiflexed, to decrease the resistance of the fibular collateral ligament and the popliteal tendon, subsequently applying direct pressure on the fibular head until the audible sensation of reduction is achieved, as was done in the present case [8-11]. In chronic cases of DPTJ, ligament reconstruction, arthrodesis, and resection of the fibular head are recommended [7,10,11].

Controversial information is observed in the scientific literature regarding follow-up and confirmation of dislocation reduction. There are cases in which the patient spontaneously reports pain improvement, after a clicking sensation (clunk) and does not consent to control imaging [1,13]. Additionally, cases have been reported in which joint stability is clinically verified without post-reduction imaging [7,12] and other cases in which it is verified with radiographs after the procedure [9]. In the present case, the reduction was verified with the absence of pain, clinical improvement, and manual evaluation of the stability of the PTJ.

Regarding the periods of immobilization and load restriction, there are authors who support immediate loading with a soft bandage and others who defend the use of immobilization with a brace for up to six weeks, with a subsequent soft bandage and support restriction for six weeks [9]. In the present case, after three weeks of immobilization with a soft bandage and protected support with crutches, a favorable clinical evolution was obtained.

## Conclusions

DPTJ is a rare injury that should have a high index of suspicion based on the kinematics of the trauma and the population group. Moreover, it is important to note that plain radiographs are valuable as an initial approach, but this pathology should not be ruled out using plain radiographs alone. On the other hand, the bibliography is scarce in this regard, which makes it difficult to make decisions on crucial elements such as confirmation of the reduction, subsequent management, and the necessary follow-up. However, the literature is consistent in showing favorable results regardless of the previously chosen method.
